# Antibiotic practice and stewardship in the management of neutropenic fever: A survey of US institutions

**DOI:** 10.1017/ash.2023.247

**Published:** 2023-09-29

**Authors:** Swarn Arya, Xiao Wang, Sonal Patel, Stephen Saw, Mary Decena, Rebecca Hirsh, David Pegues, Matthew Ziegler

## Abstract

**Background:** Neutropenic fever management decisions are complex and result in prolonged duration of broad-spectrum antibiotics. Strategies for antibiotic stewardship in this context have been studied, including de-escalation of antibiotics prior to resolution of neutropenia, with unclear implementation. Here, we present the first survey study to describe real-world neutropenic fever management practices in US healthcare institutions, with particular emphasis on de-escalation strategies after initiation of broad-spectrum antibiotics. **Methods:** Using REDCap, we conducted a survey of US healthcare institutions through the SHEA Research Network (SRN). Questions pertained to antimicrobial prophylaxis and supportive care in the management of oncology patients and neutropenic fever management (including specific antimicrobial choices and clinical scenarios). Hematologic malignancy hospitalization (2020) and bone-marrow transplantation (2016–2020) volumes were obtained from CMS and Health Resources & Services Administration databases, respectively. **Results:** Overall, 23 complete responses were recorded (response rate, 35.4%). Collectively, these entities account for ~11.0% of hematologic malignancy hospitalizations and 13.3% bone marrow transplantations nationwide. Of 23 facilities, 19 had institutional guidelines for neutropenic fever management and 18 had institutional guidelines for prophylaxis, with similar definitions for neutropenic fever. Firstline treatment universally utilized antipseudomonal broad-spectrum IV antibiotics (20 of 23 use cephalosporin, 3 of 23 use penicillin agent, and no respondents use carbapenem). Fluoroquinolone prophylaxis was common for leukemia induction patients (18 of 23) but was mixed for bone-marrow transplantation (10 of 23). We observed significant heterogeneity in treatment decisions. For stable neutropenic fever patients with no clinical source of infection identified, 13 of 23 respondents continued IV antibiotics until ANC (absolute neutrophil count) recovery. The remainder had criteria for de-escalation back to prophylaxis prior to this (eg, a fever-free period). Respondents were more willing to de-escalate prior to ANC recovery in patients with identified clinical sources (14 of 23 de-escalations in patients with pneumonia) or microbiological sources (15 of 23 de-escalations in patients with bacteremia) after dedicated treatment courses. In free-text responses, several respondents described opportunities for more systemic de-escalation for antimicrobial stewardship in these scenarios. **Conclusions:** Our results illustrate the real-world management of neutropenic fever in US hospitals, including initiation of therapy, prophylaxis, and treatment duration. We found significant heterogeneity in de-escalation of empiric antibiotics relative to ANC recovery, highlighting a need for more robust evidence for and adoption of this practice.

**Disclosures:** None

